# Herbal Medication, Macmoondong Decoction, Attenuates LPS-Induced COPD in Small Airways via TGF-*β*, CCL-2, and CXCL1

**DOI:** 10.1155/2020/6413491

**Published:** 2020-04-21

**Authors:** Soon-Young Lee, Bossng Kang, Chun-Sik Bae, Seung-Sik Cho, Dae-Hun Park

**Affiliations:** ^1^College of Korean Medicine, Dongshin University, Naju, Jeonnam 58245, Republic of Korea; ^2^Department of Emergency Medicine, College of Medicine, Hanyang University, Guri, Gyunggi 11923, Republic of Korea; ^3^College of Veterinary Medicine, Chonnam National University, Gwangju 61186, Republic of Korea; ^4^College of Pharmacy, Mokpo National University, Muan, Jeonnam 58554, Republic of Korea

## Abstract

Chronic obstructive pulmonary disease (COPD) is an incurable disease related to the respiratory system. A 2017 report by the World Health Organization stated that it was the third most common cause of death in 2015. Macmoondong decoction is a prescription that has been used widely in Korea for the treatment of respiratory diseases, but there have been few investigations into the therapeutic mechanism. To investigate the anti-COPD effect of macmoondong decoction, the animals were divided into five treatment groups: control; COPD-induced control; Spiriva; 150 mg/kg macmoondong decoction; and 1500 mg/kg macmoondong decoction. Changes typically observed in COPD, such as the populations of WBC and neutrophils in BALF, the level of IgE in serum, morphological changes, the DNA levels, and the protein expression of cytokines and chemokines (TGF-*β*, CCL-2, CXCL1, and CXCL11) in the pulmonary system, were evaluated. Macmoondong decoction inhibited the populations of WBC and neutrophils in BALF and the level of IgE in serum. Dose-dependent prevention of the pulmonary morphological changes, such as emphysema and airway fibrosis, was observed. Macmoondong decoction suppressed the expression of DNA and proteins related to the occurrence of COPD, such as TGF-*β*, CCL-2, CXCL1, and CXCL11. In particular, the expression of TGF-*β*, CCL-2, and CXCL1 was significantly suppressed by 1500 mg/kg macmoondong decoction treatment compared with Spiriva treatment. Macmoondong decoction exerted an anti-COPD effect, and the mechanism of its action may be the suppression of TGF-*β*, CCL-2, CXCL1, and CXCL11 expression, which occurred in a dose-dependent manner. The mechanism of action of macmoondong decoction may be the dose-dependent suppression of TGF-*β*, CCL-2, CXCL1, and CXCL11, with TGF-*β*, CCL-2, and CXCL1 as the potential key factors involved in COPD suppression.

## 1. Introduction

In December 2017, the World Health Organization (WHO) reported that chronic obstructive pulmonary disease (COPD) was the third most common cause of death globally, responsible for approximately 5% of total deaths [1]. Patients with COPD typically undergo changes in the respiratory system, such as neutrophil infiltration near bronchoalveolar spaces [2, 3], mucous hypersecretion [4], fibrosis around the small airways [5], and alveolar wall destruction (emphysema) [6]; these changes can lead to gas exchange abnormalities, apnea, and, ultimately, death [7].

A major causal factor for COPD is tobacco smoke, which induces an inflammatory response in the pulmonary system [8, 9]. Although the predominant stimulus for COPD is tobacco smoke, there are several irritants, such as occupational exposure and air pollution, and it occurs more commonly in individuals older than 40 years [10, 11]. In the pulmonary system, inflammation occurs in the early stages of COPD; in the next stage, a series of typical changes are observed, including goblet cell hyperplasia, mucous hypersecretion, smooth muscle hypertrophy, and fibrosis, and in the last stage, breathlessness leads to death [12, 13]. Neutrophils have a crucial role in the exacerbation of COPD, including in emphysema and mucous hypersecretion [14].

A series of cytokines and chemokines modulate COPD, including IL-8, TNF-*α*, IFN-*γ*, TGF-*β*, CCL-2, CXCL1, CXCL9, CXCL10, and CXCL11; these are related to the changes typically observed in COPD [15]. IL-8 is released by TNF-*α* stimulation, increasing the population of neutrophils [16] and finally leading to alveolar wall destruction caused by proteases such as elastase that are secreted by neutrophils [17]. IFN-*γ* stimulates epithelial cells and macrophages in the respiratory system to release various chemokines, such as CXCL9, CXCL10, and CXCL11, inducing proteases [15]. Fibrosis around the small airways is caused by TGF-*β* activation [12, 13]. CCL-2 induces monocyte recruitment; these are converted to macrophages in the lungs [18] and release CXCL1 to destroy the small airway wall [15].

As COPD is an incurable disease in which the patients' lungs are irreversibly damaged [6], conventional therapies that suppress the symptoms of COPD are commonly used. However, these result in severe adverse effects, such as airway wall thickness through bronchodilation [19], growth suppression in children, hypertension, and peptic ulcers, because of the use of corticosteroids [20]. Recently, to avoid the adverse effects of chemical drugs, new curing methods have been developed such as electroacupuncture [21, 22].

According to the Dongui Bogam, which is the clinical encyclopedia published in 1610 written by the clinician Huh [23] in Korea, some prescriptions have been used for therapeutic purposes for respiratory diseases, such as macmoondong decoction, sochungryong decoction, chungsangbohwahwan, and samsoyum; however, no evidence has been established for the mode of action. Although macmoondong decoction has been used widely as a traditional prescription for pulmonary diseases and, according to the Dongui Bogam [23], comprises *Glycyrrhizae radix*, *Oryza sativa*, *Ziziphus jujuba* inermis, *Ophiopogon japonicus*, *Pinellia ternate*, and *Panax ginseng*, the therapeutic mechanism has not been elucidated.

In this study, the therapeutic effect and mechanism of macmoondong decoction were investigated by using an LPS-induced COPD model.

## 2. Materials and Methods

### 2.1. Material

Macmoondong decoction is an over-the-counter (OTC) medication manufactured by Hankuk Inspharm, Ltd. (Jeonnam, Korea) and produced in accordance with the prescription of macmoondong decoction; the Dongui Bogam [23] indicates that macmoondong decoction should consist of *G. radix*, *O. sativa*, *Z*. *jujuba inermis*, *O. japonicas*, *P. ternate*, and *P. ginseng*.

### 2.2. Animal Experiment

**Figure 1:**
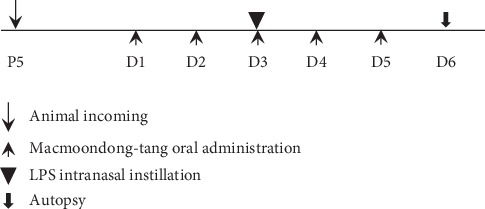
The schedule of animal experiments for the investigation of the COPD therapeutic effect and the anti-COPD mechanism of macmoondong decoction.

The animal experiments were conducted based on modifications of Kobayashi's method [24]. In our previous study, we established a COPD model at 72 h after LPS intranasal instillation [25]. The same experiments were conducted twice to ensure reproducibility, with 35 mice used per experiment. Seventy 4-week-old female BALB/c mice were purchased from Samtako (Osan, Korea) and acclimatized to the animal facility for 7 days. As shown in Figure 1, the animals were divided into five groups based on the treatment agent: the control group, administered saline without LPS treatment for 5 days; the COPD induction group, administered a single intranasal instillation of 0.8 mg/kg LPS; the positive control group, administered 1 mg/kg Spiriva (NDC 0597-0075-41, Boehringer Ingelheim Pharmaceuticals Inc., Ridgefield, CT, USA) for 5 days and the single LPS administration; 150 mg/kg macmoondong decoction group, administered 150 mg/kg macmoondong decoction treatment for 5 days and the single LPS administration; and the 1500 mg/kg macmoondong decoction group, administered 1500 mg/kg macmoondong decoction treatment for 5 days and the single LPS administration. The macmoondong decoction dose in animals was determined based on the recommended therapeutic dose in humans. All agents were administered via the oral route, except for LPS treatment, which was administered as an intranasal instillation. On day 3, after each agent treatment, 0.8 mg/kg LPS was instilled through the intranasal route, and on day 6, all animals were anesthetized with 50 mg/kg Zoletil (Virbac, Carros, France).

### 2.3. Bronchoalveolar Fluid (BALF) and Serum Analysis

BALF and serum analysis was conducted as described in our previous study [26]. The animal study was conducted twice to verify reproducibility. In the first study, three mice were used for the evaluation of BALF and serum, with the remaining four mice used for other studies, such as the morphological analysis (H&E stain and Masson's trichrome stain) and the expression of specific proteins related to the occurrence of COPD (IHC). BALF collection was scheduled from all mice after anesthetization with 50 mg/kg Zoletil (Virbac, Carros, France), and the tracheas were cannulated with disposable animal feeding needles. Lavage was performed with three 0.4 mL aliquots of cold phosphate-buffered saline (PBS), and BALF samples were collected and immediately centrifuged at 3,000 rpm for 5 min (Sorvall Legend Micro 17R, Thermo Fisher Scientific Inc., Waltham, MA, USA). The cell pellets were resuspended in PBS, and total and differential cell counts were obtained. The number of total cells and differential cells was counted with a Hemavet Multispecies Hematology System (Drew Scientific Inc., Waterbury, CT, USA). After cell collection (if used), the animals were sacrificed by an additional Zoletil injection. IgE levels in the serum were measured using a specific mouse IgE ELISA kit (BD Bioscience, catalog number 555248, San Jose, CA, USA) in accordance with the manufacturer's protocols.

### 2.4. Histopathological Analysis

Histopathological analysis was conducted as previously described [27]. Lung tissues were fixed in 10% (v/v) formaldehyde solution, dehydrated in a graded ethanol series (99.9%, 90%, 80%, and 70%), and embedded in paraffin. In total, eight animals subjected to a particular treatment condition were studied over both studies, with four animals per group used for histological analysis. Lung tissues were sectioned into 4 *μ*m longitudinal slices and stained with H&E and Masson's trichrome.

### 2.5. Real-Time Polymerase Chain Reaction (q-PCR)

To evaluate the change in cDNA expression of TGF-*β*, CCL-2, CXCL1, and CXCL11, which are related to COPD occurrence, the total RNA was extracted from the lungs by using the RNeasy Mini Kit (Qiagen, Hilden, Germany) in accordance with the manufacturer's instructions. Total RNA (100 ng) was used as a template for the reaction, with the following primers synthesized for q-PCR:

  TGF-*β* forward 5′-CTTCAGCTCCACAGAGAAGAACTGC-3′
  TGF-*β* reverse 5′-CACAATCATGTTGGACAACTGCTCC-3′
  CCL-2 forward 5′-AACTCTCACTGAAGCCAGCTCT-3′
  CCL-2 reverse 5′-CGTTAACTGCATCTGGCTGA-3′
  CXCL1 forward 5′-ATCCAGAGCTTGAAGGTGTTG-3′
  CXCL1 reverse 5′-GTCTGTCTTCTTTCTCCGTTACTT-3′
  CXCL11 forward 5′-CTGCTCAAGGCTTCCTTATGTT-3′
  CXCL11 reverse 5′-CCTTTGTCGTTTATGAGCCTTC-3′
  GAPDH forward 5′-GTGGAGTCATACTGAACATGTAG-3′
  GAPDH reverse 5′-AATGGTGAAGGTCGGTGTG-3′

The q-PCR conditions comprised denaturation at 95°C for 5 s, followed by 40 cycles of annealing/extension at 65°C for 30 s by using QTOWER 2.2 (Analytik Jena AG, Thuringia, Germany).

### 2.6. Immunohistochemical (IHC) Analysis

IHC analysis was conducted as previously described [27]. Deparaffinized tissue sections were treated with 3% hydrogen peroxide in methanol for 10 min to remove endogenous peroxidase. Antigen retrieval was performed in sodium citrate buffer (0.1 M) by using a microwave method. The slides were incubated with normal serum to block nonspecific binding and then incubated for 1 h with primary antibodies (1 : 100 to 1 : 200 dilutions) to TGF-*β* (MBS462142, MyBioSource), CCL-2 (PAB16617, Abnova, Taipei, Taiwan), CXCL1 (PAB8798, Abnova), and CXCL11 (bs-2552R, Bioss). The slides were incubated for 10 min with biotinylated secondary antibodies (PK-7800, Vector Laboratories, Burlingame, CA, USA) and horseradish peroxidase-conjugated streptavidin. The signals were detected by the application of the 3,3-diaminobenzidine tetrahydrochloride substrate chromogen solution and counterstaining with Mayer's hematoxylin. To evaluate the staining, after five circles of equal diameter had been drawn on separate areas, without overlapping, the positive cells were counted from four slides per group.

### 2.7. Statistical Analysis

The results are expressed as the mean ± standard deviation (SD). The differences between groups were evaluated by using one-way analysis of variance, followed by Dunnett's multiple comparison test. *P* values of <0.01 and <0.05 were considered to indicate statistical significance.

## 3. Results

### 3.1. Macmoondong Decoction Suppressed Increases in WBC and Neutrophils in BALF and IgE in Serum

**Figure 2:**
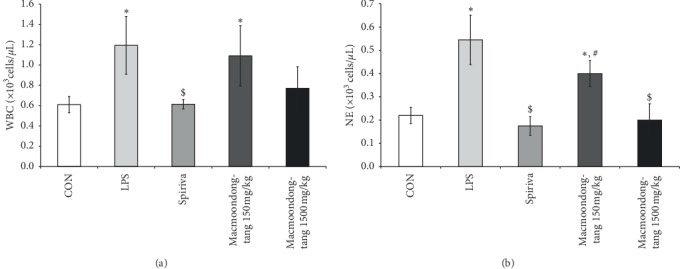
Macmoondong decoction exerted the dose-dependent suppression of the WBC population and significantly inhibited neutrophil proliferation in the LPS-induced intranasal instillation model of COPD. (a) Macmoondong decoction dose-dependently inhibited the WBC population in BALF. (b) Neutrophil proliferation was significantly suppressed by macmoondong decoction treatment in a dose-dependent manner. Each bar represents the mean ± SD (*N* = 6). ^*∗*^*P* < 0.05 vs. control group; ^$^*P* < 0.05 vs. COPD induction group; ^#^*P* < 0.05 vs. Spiriva treatment group.

**Figure 3:**
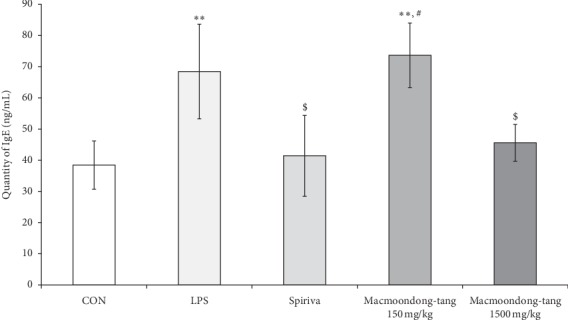
Macmoondong decoction significantly downregulated the level of IgE in serum. Each bar represents the mean ± SD (*N* = 6). ^*∗∗*^*P* < 0.01 vs. control group; ^$^ *P* < 0.05 vs. COPD induction group; ^#^*P* < 0.05 vs. Spiriva treatment group.

Generally, patients with COPD experience an increase in the population of neutrophils [28]; thus, the change in neutrophil level is an important biomarker for the evaluation of the severity of COPD. To measure the population of neutrophils after the oral administration of macmoondong decoction for 5 days, BALF was collected from all experimental animals. However, there was no statistically significant difference between the 150 mg/kg macmoondong decoction treatment and the 1500 mg/kg macmoondong decoction treatment in the populations of WBC (Figure 2(a)), which were highest in the LPS treatment group and lowest in the Spiriva treatment group. Macmoondong decoction might have therefore caused dose-dependent suppression of WBC in the LPS-induced murine model. Macmoondong decoction effectively inhibited neutrophil proliferation by LPS in a dose-dependent manner (Figure 2(b)). IgE is a very important biomarker for the analysis of the severity of COPD, as the level of IgE was significantly upregulated in patients with COPD [29]. To estimate the level of IgE in the serum, an ELISA was performed. The effect of macmoondong decoction on IgE was very similar to the effect on neutrophils (Figures 2 and 3); in addition, LPS treatment effectively induced IgE level, which was suppressed in the Spiriva treatment group. Macmoondong decoction decreased the level of IgE in a dose-dependent manner.

### 3.2. Macmoondong Decoction Effectively Suppressed the Typical Changes Caused by COPD in the Lung

**Figure 4:**
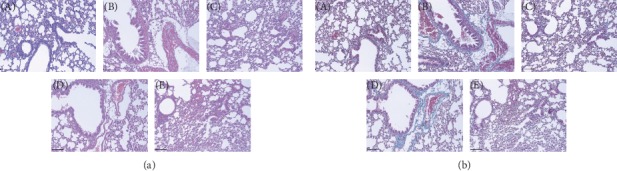
Macmoondong decoction inhibited the typical changes observed in COPD. (a) In the H&E-stained sections of the lung, macmoondong decoction suppressed the morphological changes related to COPD, such as mucous hypersecretion and inflammatory cell infiltration near the small airways. (b) In Masson's trichrome-stained sections of the lung, macmoondong decoction dose-dependently prevented fibrogenicity. *N* = 6. Scale bar =100 *μ*m. (A) control; (B) 0.8 mg/kg COPD induction group; (C) 1 mg/kg Spiriva treatment for 5 days after a single LPS instillation; (D) 150 mg/kg macmoondong decoction treatment for 5 days after a single LPS instillation; (E) 1500 mg/kg macmoondong decoction treatment for 5 days after a single LPS instillation.

In the lungs of patients with COPD, several typical morphological changes are observed, such as fibrosis, alveolar destruction (emphysema), and inflammatory cell infiltration [13]. To measure the suppressive effect of macmoondong decoction on the morphological changes induced by LPS treatment, H&E staining (Figure 4(a)) and Masson's trichrome (Figure 4(b)) were conducted. In the COPD induction group (Figures 4(a) (B) and 4(b) (B)), typical morphological changes, such as mucous hypersecretion, inflammatory cell infiltration, alveolar wall destruction, and fibrosis, were observed. Macmoondong decoction dose-dependently suppressed mucous hypersecretion, inflammatory infiltration, and alveolar wall destruction (Figures 4(a) (D) and 4(a) (D) and (E)), as well as fibrosis around the small airways (Figures 4(b) (D) and (E)).

### 3.3. Macmoondong Decoction Significantly Downregulated the DNA Expression of Cytokine/Chemokine Related to COPD Occurrence

**Figure 5:**
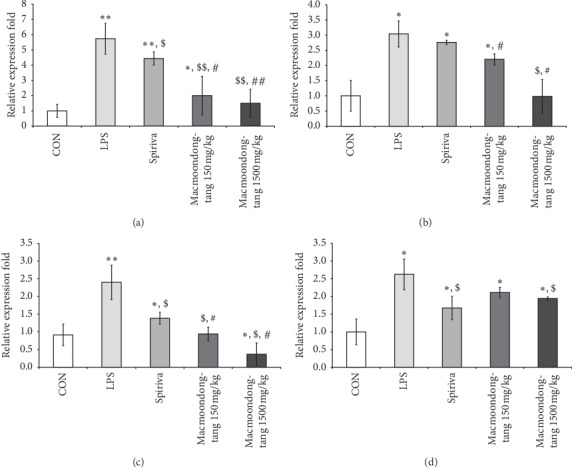
Macmoondong decoction dose-dependently suppressed the DNA expression of TGF-*β*, CCL-2, CXCL1, and CXCL11. (a) The DNA expression of TGF-*β* in the macmoondong decoction-treated groups was lower than that in the Spiriva treatment group; in addition, a dose-dependent suppression was observed. (b) The change in DNA expression of CCL-2 was similar to the pattern in TGF-*β*. (c) Macmoondong decoction effectively downregulated the DNA expression of CXCL1; the pattern of changes was very similar to that of TGF-*β*. (d) Compared with the changes in other genes, such as TGF-*β*, CCL-2, and CXCL1, small changes were observed, but macmoondong decoction effectively suppressed the DNA expression of CXCL11. Each bar represents the mean ± SD (*N* = 8). ^*∗*^*P* < 0.05 vs. control group; ^*∗∗*^*P* < 0.01 vs. control group; ^$^ *P* < 0.05 vs. COPD induction group; ^$$^*P* < 0.01 vs. COPD induction group; ^#^*P* < 0.05 vs. Spiriva treatment group; ^#^*P* < 0.05 vs. Spiriva treatment group.

We have previously reported the relationship between cytokines and chemokines and COPD severity [27]. In this study, the expressions of COPD-related cytokine genes IL-8 and TGF-*β* and chemokine genes CCL-2, CXCL1, CXCL9, CXCL10, and CXCL11 were measured. However, only the expression of TGF-*β*, CCL-2, CXCL1, and CXCL11 was found to be altered (Figure 5). The changes in DNA expression of TGF-*β*, CCL-2, and CXCL1 were very similar, but there were differences between the DNA expression of CXCL11 and the other genes. The DNA expression of TGF-*β*, CCL-2, and CXCL1 in both the 150 mg/kg macmoondong decoction and the 1500 mg/kg macmoondong decoction groups was lower than that in the Spiriva treatment group (Figures 5(a) and 5(c)); however, as shown in Figure 5(d), the DNA expression of CXCL11 in the Spiriva treatment group was lower than those in the macmoondong decoction treatment groups. These results indicate that macmoondong decoction may be more effective than Spiriva for the regulation of TGF-*β*, CCL-2, and CXCL1.

### 3.4. Macmoondong Decoction Effectively Suppressed Some Cytokines and Chemokines in the LPS-Induced COPD Model in a Dose-dependent Manner

**Figure 6:**
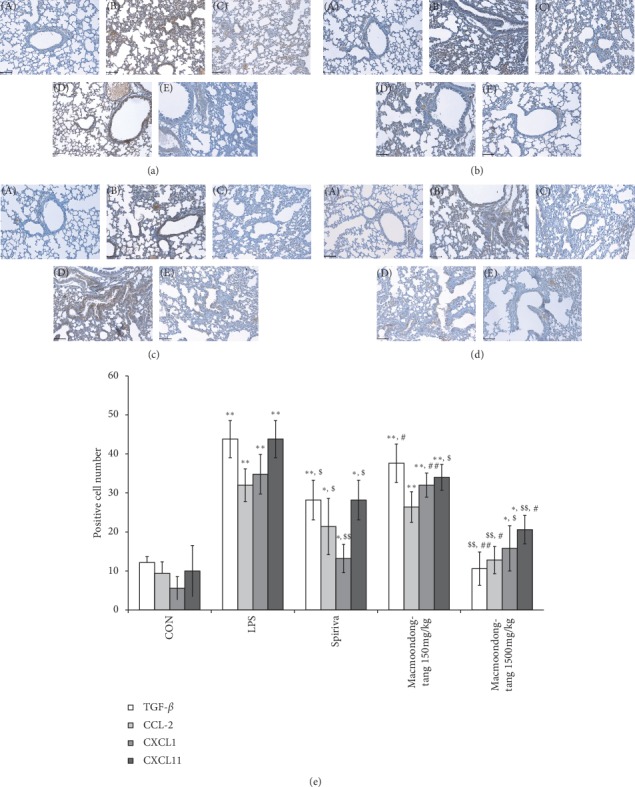
Macmoondong decoction dose-dependently suppressed the expression of TGF-*β*, CCL-2, CXCL1, and CXCL11. (a) In the 1500 mg/kg macmoondong decoction treatment group, the expression of TGF-*β* was lower than that in the Spiriva treatment group, and dose-dependent inhibition was observed. (b) Macmoondong decoction significantly inhibited the expression of CCL-2 in a dose-dependent manner, and in the 1500 mg/kg macmoondong decoction treatment group, the suppression of CCL-2 was more effective than that in Spiriva treatment. (c) Macmoondong decoction dose-dependently downregulated the expression of CXCL1. (d) Similar to the changes in CXCL1, macmoondong decoction exerted dose-dependent suppression. (e) The positively stained cell counts for TGF-*β*, CCL-2, CXCL1, and CXCL11. Each bar represents the mean ± SD (*N* = 8). ^#^*P* < 0.05 vs. control group; ^*∗∗*^*P* < 0.01 vs. control group; ^$^ *P* < 0.05 vs. COPD induction group; ^$$^*P* < 0.01 vs. COPD induction group; ^#^*P* < 0.05 vs. Spiriva treatment group; ^##^*P* < 0.01 vs. Spiriva treatment group.

The expression of TGF-*β* was dose-dependently decreased by macmoondong decoction treatment; in particular, the expression in the 1500 mg/kg group was lower than that in the Spiriva treatment group (Figure 6(a)). Although the expression of CCL-2 was downregulated by Spiriva treatment, the expression was higher than that in the control group (Figure 6(b)). However, in the 1500 mg/kg macmoondong decoction treatment group, the expression of CCL-2 was suppressed to a level similar to that in the control group, which indicated that the suppressive effect of macmoondong decoction on CCL-2 expression was higher than that of Spiriva. The effect of macmoondong decoction on CXCL1 expression was dose-dependent, with the expression in the 1500 mg/kg macmoondong decoction group at a similar level to that of the Spiriva group (Figure 6(c)). However, macmoondong decoction did not completely suppress the expression of CXCL1. The modulatory effect of macmoondong decoction on CXCL11 was slightly different from the other genes, such as TGF-*β*, CCL-2, and CXCL1 (Figure 6(d)). The expression of CXCL11 was downregulated not only by macmoondong decoction, but also by Spiriva, and the change induced by 1500 mg/kg macmoondong decoction treatment was greater than that induced by Spiriva treatment. However, there was little difference between the suppressive effects of macmoondong decoction and Spiriva.

## 4. Discussion

Globally, the number of deaths from COPD is very high; in 2017, the WHO reported that, worldwide, COPD was the third most common cause of death [1]. However, it is of greater concern that COPD may still be the third most common cause of death in 2030 [30]. The severity of COPD is of serious concern relative to the age of society, as the rate of occurrence is higher in older individuals than in younger individuals, and globally, the average age of societies is increasing. In particular, there are many opportunities through which people can come into contact with the COPD irritants, such as tobacco smoke, air pollution, and occupational dusts and fumes; subsequently, COPD onset in the pulmonary system is irreversible [6] and this disease leads to severe problems. At present, the drugs for COPD treatment, including bronchodilators and corticosteroids, have many adverse effects [19, 20]. For several decades, many trials have been attempted to find anti-COPD drugs with greater efficacy and lower toxicity [31].

Macmoondong decoction has been used as a traditional medicine for respiratory diseases and its prescription was declared in the Dongui Bogam [23]. Macmoondong decoction is a mixture of plant medicine extracts used in traditional Korean medicine; however, as there were no previous reports that investigated the mode of action, we elected to study the anti-COPD mechanism.

In patients with COPD, representative changes in the pulmonary system include mucous hypersecretion [4], fibrosis [5], and alveolar wall destruction (emphysema) [6]; these changes are related to several factors such as cytokines (TGF-*β*, TNF-*α*, IL-1*β*, and IL-6), chemokines (CCL2, CXCL1, and CCR2), and collagenase/gelatinase (MMP-9 and MMP-12) [15, 32]. TGF-*β* is related to the fibrosis around the small airways and is released from pulmonary epithelial cells [17]; in our results, macmoondong decoction dose-dependently suppressed the fibrogenicity near the alveoli (Figure 4(b) (D) and 4(b) (E)) via the downregulation of TGF-*β* expression (Figures 5(a) and 6(a)); in particular, in the 1500 mg/kg macmoondong decoction treatment group, the expression of TGF-*β* was lower than that in the Spiriva treatment group. CCL-2 stimulates the recruitment of monocytes, which are changed to macrophages in the lung parenchyma; through these changes in macrophages, CXCL1 release was induced, which stimulated neutrophil activation to enhance the alveolar wall destruction through the control of elastase secretion [15, 18]. In Figures 5(b) and 6(b), the expression of CCL-2 was effectively decreased by macmoondong decoction treatment; in Figures 5(c) and 6(c), the changes in CXCL1 expression were similar to the changes in CCL-2, and finally, macmoondong decoction dose-dependently inhibited the alveolar wall destruction (Figures 4(a) (D) and (E)). The decrease in CCL-2 and CXCL1 expression sequentially induced a decrease in neutrophil population (Figure 2(b)) and reduced the occurrence of emphysema (Figures 4(a) (D) and 4(a) (E)). CXCL11, which is secreted by the recruited macrophages, stimulated the activation of Th1/Tc cells and induced chronic inflammation and emphysema [33]; macmoondong decoction significantly suppressed the expression of CXCL11 to similar levels induced by Spiriva treatment (Figures 5(d) and 6(d)). Indeed, even in the 1500 mg/kg macmoondong decoction treatment group, the CXCL11 expression was lower than that induced by Spiriva treatment.

To find the anti-COPD mechanism of macmoondong decoction in the LPS-induced COPD model, we evaluated the functional changes, morphological changes, and the alteration of COPD-related cytokines and chemokines.

Our results indicated that macmoondong decoction suppressed the typical changes induced by LPS treatment, such as increases in WBC and neutrophil counts in BALF and IgE in serum, morphological changes in the lung (mucous hypersecretion, inflammatory cell infiltration, alveolar wall destruction, and small airway fibrosis), DNA upregulation of certain cytokines and chemokines (TGF-*β*, CCL-2, CXCL1, and CXCL11) in the pulmonary system, and increases in their proteins (TGF-*β*, CCL-2, CXCL1, and CXCL11) in the respiratory system. In particular, in the 1500 mg/kg macmoondong decoction treatment group, one cytokine (TGF-*β*) and two chemokines (CCL-2 and CXCL1) were suppressed to a greater extent than those in the Spiriva treatment group.

In this study, we concluded that macmoondong decoction was an effective prescription for COPD therapy via the suppression of TGF-*β*, CCL-2, and CXCL1.

## 5. Conclusions

Macmoondong decoction, which has been used as a traditional medicine against respiratory diseases, is an anti-COPD drug with effective therapeutic function. The mechanism of action of macmoondong decoction may be the dose-dependent suppression of TGF-*β*, CCL-2, CXCL1, and CXCL11, with TGF-*β*, CCL-2, and CXCL1 as the potential key factors involved in COPD suppression.

## Data Availability

All of the data used to support the findings of this study could be freely available to be used under no restriction.

## References

[1] World Health Organization (2017). *Chronic Obstructive Pulmonary Disease (COPD)*.

[2] Pesci A., Majori M., Cuomo A. (1998). Neutrophils infiltrating bronchial epithelium in chronic obstructive pulmonary disease. *Respiratory Medicine*.

[3] O’Donnell R. A., Peebles C., Ward J. A. (2004). Relationship between peripheral airway dysfunction, airway obstruction, and neutrophilic inflammation in COPD. *Thorax*.

[4] Ramos F. L., Krahnke J. S., Kim V. (2014). Clinical issues of mucus accumulation in COPD. *International Journal of Chronic Obstructive Pulmonary Disease*.

[5] Nowrin K., Sohal S. S., Peterson G., Patel R., Walters E. H. (2014). Epithelial-mesenchymal transition as a fundamental underlying pathogenic process in COPD airways: fibrosis, remodeling and cancer. *Expert Review of Respiratory Medicine*.

[6] Aldonyte R., Bagdonas E., Raudoniute J., Bruzauskaite I. (2015). Novel aspects of pathogenesis and regeneration mechanisms in COPD. *International Journal of Chronic Obstructive Pulmonary Disease*.

[7] O’Byrne P. M. (2007). Exacerbations of asthma and COPD: definitions, clinical manifestations and epidemiology. *Contributions to Microbiology*.

[8] Franklin W., Lowell F. C., Michelson A. L., Schiller I. W. (1956). Chronic obstructive pulmonary emphysema; a disease of smokers. *Annals of Internal Medicine*.

[9] Ludwig P. W., Schwartz B. A., Hoidal J. R., Niewoehner D. E. (1985). Cigarette smoking causes accumulation of polymorphonuclear leukocytes in alveolar septum. *The American Review of Respiratory Disease*.

[10] Forbes L. J. L., Kapetanakis V., Rudnicka A. R. (2009). Chronic exposure to outdoor air pollution and lung function in adults. *Thorax*.

[11] Hopkinson N. S., Polkey M. I. (2009). Chronic obstructive pulmonary disease in non-smokers. *The Lancet*.

[12] Hogg J. C., Chu F., Utokaparch S. (2004). The nature of small-airway obstruction in chronic obstructive pulmonary disease. *New England Journal of Medicine*.

[13] Hogg J. C., Timens W. (2009). The pathology of chronic obstructive pulmonary disease. *Annual Review of Pathology: Mechanisms of Disease*.

[14] Butler A., Walton G. M., Sapey E. (2018). Neutrophilic inflammation in the pathogenesis of chronic obstructive pulmonary disease. *COPD: Journal of Chronic Obstructive Pulmonary Disease*.

[15] Barnes P. J. (2009). The cytokine network in chronic obstructive pulmonary disease. *American Journal of Respiratory Cell and Molecular Biology*.

[16] Keatings V. M., Collins P. D., Scott D. M., Barnes P. J. (1996). Differences in interleukin-8 and tumor necrosis factor-alpha in induced sputum from patients with chronic obstructive pulmonary disease or asthma. *American Journal of Respiratory and Critical Care Medicine*.

[17] Barnes P. J. (2008). Immunology of asthma and chronic obstructive pulmonary disease. *Nature Reviews Immunology*.

[18] de Boer W. I., Sont J. K., van Schadewijk A., Stolk J., van Krieken J. H., Hiemstra P. S. (2000). Monocyte chemoattractant protein 1, interleukin 8, and chronic airways inflammation in COPD. *The Journal of Pathology*.

[19] Kim V., Desai P., Newell J. D. (2014). Airway wall thickness is increased in COPD patients with bronchodilator responsiveness. *Respiratory Research*.

[20] McEvoy C. E., Niewoehner D. E. (1997). Adverse effects of corticosteroid therapy for COPD. *Chest*.

[21] Zhang X.-f., Xiang S.-y., Geng W.-y. (2018). Electro-acupuncture regulates the cholinergic anti-inflammatory pathway in a rat model of chronic obstructive pulmonary disease. *Journal of Integrative Medicine*.

[22] Zhang X.-f., Zhu J., Geng W.-y. (2014). Electroacupuncture at Feishu (BL13) and Zusanli (ST36) down-regulates the expression of orexins and their receptors in rats with chronic obstructive pulmonary disease. *Journal of Integrative Medicine*.

[23] Jun H. (2002). *Dongui Bogam*.

[24] Kobayashi S., Fujinawa R., Ota F. (2013). A single dose of lipopolysaccharide into mice with emphysema mimics human chronic obstructive pulmonary disease exacerbation as assessed by micro-computed tomography. *American Journal of Respiratory Cell and Molecular Biology*.

[25] Lee S.-Y., Cho J.-H., Cho S. S., Bae C.-S., Kim G.-Y., Park D.-H. (2018). Establishment of a chronic obstructive pulmonary disease mouse model based on the elapsed time after LPS intranasal instillation. *Laboratory Animal Research*.

[26] Lee S.-Y., Bae C.-S., Choi Y.-h. (2017). Opuntia humifusamodulates morphological changes characteristic of asthma via IL-4 and IL-13 in an asthma murine model. *Food & Nutrition Research*.

[27] Lee S. Y., Bae C. S., Seo N. S. (2019). *Camellia japonica* oil suppressed asthma occurrence via GATA-3 & IL-4 pathway and its effective and major component is oleic acid. *Phytomedicine*.

[28] Gompertz S., O’Brien C., Bayley D. L., Hill S. L., Stockley R. A. (2001). Changes in bronchial inflammation during acute exacerbations of chronic bronchitis. *European Respiratory Journal*.

[29] Samaha H. M. S., Elsaid A. R., NasrEldin E. (2015). Total serum IgE level in COPD patients. *Egyptian Journal of Chest Diseases and Tuberculosis*.

[30] Mathers C. D., Loncar D. (2006). Projections of global mortality and burden of disease from 2002 to 2030. *PLoS Medicine*.

[31] Pharmaceutical Research and Manufacturers of America (2012). *Medicines in Development COPD 2012*.

[32] Linder R., Rönmark E., Pourazar J., Behndig A., Blomberg A., Lindberg A. (2015). Serum metalloproteinase-9 is related to COPD severity and symptoms—cross-sectional data from a population based cohort-study. *Respiraotry Research*.

[33] Barnes P. J. (2014). Cellular and molecular mechanisms of chronic obstructive pulmonary disease. *Clinics in Chest Medicine*.

